# Bioinformatic Challenges Detecting Genetic Variation in Precision Medicine Programs

**DOI:** 10.3389/fmed.2022.806696

**Published:** 2022-04-08

**Authors:** Matt A. Field

**Affiliations:** ^1^Centre for Tropical Bioinformatics and Molecular Biology, College of Public Health, Medical and Veterinary Science, James Cook University, Cairns, QLD, Australia; ^2^Immunogenomics Lab, Garvan Institute of Medical Research, Darlinghurst, NSW, Australia; ^3^Menzies School of Health Research, Charles Darwin University, Darwin, NT, Australia

**Keywords:** precision medicine, variant detection, high-throughput sequencing, pathogenic variant, variant prioritization, FPGA—field-programmable gate array, GPU-accelerated

## Abstract

Precision medicine programs to identify clinically relevant genetic variation have been revolutionized by access to increasingly affordable high-throughput sequencing technologies. A decade of continual drops in per-base sequencing costs means it is now feasible to sequence an individual patient genome and interrogate all classes of genetic variation for < $1,000 USD. However, while advances in these technologies have greatly simplified the ability to obtain patient sequence information, the timely analysis and interpretation of variant information remains a challenge for the rollout of large-scale precision medicine programs. This review will examine the challenges and potential solutions that exist in identifying predictive genetic biomarkers and pharmacogenetic variants in a patient and discuss the larger bioinformatic challenges likely to emerge in the future. It will examine how both software and hardware development are aiming to overcome issues in short read mapping, variant detection and variant interpretation. It will discuss the current state of the art for genetic disease and the remaining challenges to overcome for complex disease. Success across all types of disease will require novel statistical models and software in order to ensure precision medicine programs realize their full potential now and into the future.

## Introduction

Precision medicine programs are increasingly being implemented worldwide with a goal of improving patient care for an individual (1). Largely enabled by access to increasingly affordable high quality sequence data, great strides have been made in the diagnosis and management of genetic disease. By considering a patients unique genetic, environmental and lifestyle factors precision medicine aims to develop customized patient-specific treatments. Increasingly important in precision medicine programs is the ability to utilize genetic information to stratify patients with regard to treatment options and outcomes. Such patient information can be broadly classified into predicative and prognostic biomarkers with prognostic biomarkers informing on patient outcome in contrast to predictive biomarkers which directly guides treatment (the focus of this review). Currently, diagnosis and treatment of cancer and rare diseases are the largest beneficiaries of precision medicine programs. In cancer, huge numbers of druggable molecular alterations have been described and cataloged in growing public repositories like Clinical Interpretation of Variants in Cancer (CIViC) (2). As of February 2022, CIViC contain an incredible 3,041 actionable variants in 464 genes supported by 8,576 evidence items. Beyond cancer, the genetic cause of more than 80% of the roughly 6,000 known rare diseases has been elucidated in the last decade alone (3). While impressive, currently only ~5% of these diseases have an accepted targeted treatment indicative of the work still required (3).

Despite progress in diagnosing and treating genetic diseases, a bottleneck persists in variant interpretation. The increase in sequencing capacity has identified huge numbers of new suspected pathogenic variants however there is often sparse or inconclusive supporting functional evidence. For example, cystic fibrosis (CF) is caused by up to ~300 pathogenic variants in the *CTFR* gene however their impact is often heterogenous amongst individuals (4). Functional inference prediction tools are often run instead to access the likelihood of a mutation ablating protein function however such tools are known to have high false positive rates (5). Overall, substantial progress has been made in genetic disease however numerous challenges need to be addressed before precision medicine programs can be delivered at scale and for complex, polygenic disease.

Reliably identifying disease causing variants remains a challenge within the field particularly for complex disease. While great strides have been made for cancer and rare diseases, the diagnosis rates for complex diseases remain much lower (6). Despite these challenges, there are many examples of genetic traits in polygenic disease contributing to clinical manifestations [e.g., blood disease (7), autoimmune disease (8)]. To increase the diagnosis rates for complex diseases, previous approaches have employed a wide variety of strategies. For example, careful sample selection improve diagnosis rates by focusing on families with multiple affected individuals who exhibit extreme phenotypes and early onset of disease (9). Additionally, particular variant classes can be prioritized in different scenarios such as homozygous mutations for consanguineous pedigrees (10) and *de novo* mutations for trios with an affected child and unaffected parents (11). While these strategies are feasible in particular scenarios, in many cases only a single patient is available meaning prioritization strategies must consider all genetic variation detected in a patient.

An additional challenge in variant detection is the increased recognition of the importance of larger copy number and repeat variation in driving disease. These variant classes are harder to reliably detect than single nucleotide variants (SNVs) and small insertion/deletions (indels) particularly with short read sequencing technologies (12). Even for SNVs and small indels there are limitations with most precision medicine programs prioritizing variants disrupting gene function yet increasingly portions of the “missing heritability” in disease is being explained by small variants that either generate unexpected splicing errors or disrupt poorly annotated regulatory elements (13). These challenges are compounded within populations of non-European ancestry due the over representation of individuals of European ancestry within public variant databases. While this trend is improving, a 2016 study found 81% of all GWAS study samples were of European ancestry with only 4% of all samples being of African or Latin American ancestry or Indigenous (14).

Inherent to any successful precision medicine program is the timely and accurate detection of genetic variation and the prioritization of the variants most likely to be relevant to the patient's condition. Advances in software and hardware are playing an increasingly innovative role in delivering on these goals particularly for accurate variant detection and prioritization. Software-based approaches are varied and include developing new algorithms, increasing efficiencies of existing algorithms, increasing parallelization and improved standardization of common file formats (15). Hardware-based approaches are increasingly important and include increased availability of cluster and cloud based compute environments (16), field-programmable gate arrays (FPGA) devices (17) and graphical processing units (GPU) enabled bioinformatics algorithms (18).

Pharmacogenetic variants are also important in precision medicine with individual variability in drug response increasingly being attributed to genetic variation. An average individual is estimated to carry three clinically actionable pharmacogenetic variants with 97% of individuals carrying at least one such variant (19). Increasingly large repositories that aggregate and annotate pharmacogenetic variants [e.g., PharmGKB (20)] are being used in drug dosage decision making. While encouraging, the majority of known pharmacogenetic variants remain underutilized in precision medicine. This is largely due to a poor understanding of the underlying mechanisms and challenges in accurately identifying and annotating pharmacogenetic variants. For example, a recent study showed pharmacogenetic variants causing missense mutations and associated with off-target effects are incorrectly classified as benign by functional inference prediction software (21). Further software development is needed to account for this special class of variation (22).

Large-scale translation of research results into the clinic remain a significant bottleneck for the wide-spread implementation of precision medicine programs. While increasingly detailed annotation and prioritization workflows are being described and shared (23), most still remain siloed within individual institutions or are bound to specific hardware configurations. Improved containerization of workflows is helping to facilitate sharing of analysis pipelines (24) with initiatives like the Global Alliance for Genomics and Health (GA4GH) facilitating the timely sharing of large genetic data sets. While improving standardization and sharing of resources is critical, a larger challenge is the availability of accurate databases of clinically actionable variants. While many such repositories exist, studies have identified inaccuracies throughout (25). To illustrate, a recent study followed up 239 variants in the Human Gene Mutation Database (HGMD) classified as disease-causing and found only 7.5% of these variants met the criteria required to be called disease-causing (26). For precision medicine to succeed at scale, more accurate and detailed databases of clinically actionable variants are required.

Despite substantial progress, reliably detecting genetic variants within precision medicine programs has many challenges remaining. While solutions are actively being developed it is clear more improvements are needed if we are to realize the full potential of population-wide precision medicine programs. In this review, I will describe the current and future challenges for identifying clinically relevant genetic variants in precision medicine programs with resources summarized in Table 1.

**Table 1:** Resources for variant detection in precision medicine programs.

**Database**	**Function**	**Web link**
dbSNP (27)	Population level variation	http://www.ncbi.nlm.nih.gov/snp
gnomAD (28)	Population level variation	https://gnomad.broadinstitute.org
1000 Genomes Phase 3 (29)	Population level variation	http://phase3browser.1000genomes.org
Database of Genomic Variants (30)	Population level variation	http://dgv.tcag.ca/dgv/app/home
Variant Effect Predictor (31)	Variant annotation	https://ensembl.org/info/docs/tools/vep/index.html
dbNFSP (32)	Variant annotation	https://sites.google.com/site/jpopgen/dbNSFP
AnnoVar (33)	Variant annotation	http://annovar.openbioinformatics.org/en/latest/
ClinVar (34)	Clinical annotation	https://www.ncbi.nlm.nih.gov/clinvar
LOVD (35)	Clinical annotation	http://www.lovd.nl
PolyPhen2 (36)	Functional impact	http://genetics.bwh.harvard.edu/pph2/
SIFT (37)	Functional impact	https://sift.bii.a-star.edu.sg/
CADD (38)	Functional impact	https://cadd.gs.washington.edu/
GTEx (39)	Gene expression	https://gtexportal.org
Multi-symbol checker (40)	Gene naming	https://www.genenames.org/tools/multi-symbol-checker
OMIM (41)	Gene / disease annotation	https://www.omim.org

## Current Challenges and Solutions

**Figure 1:**
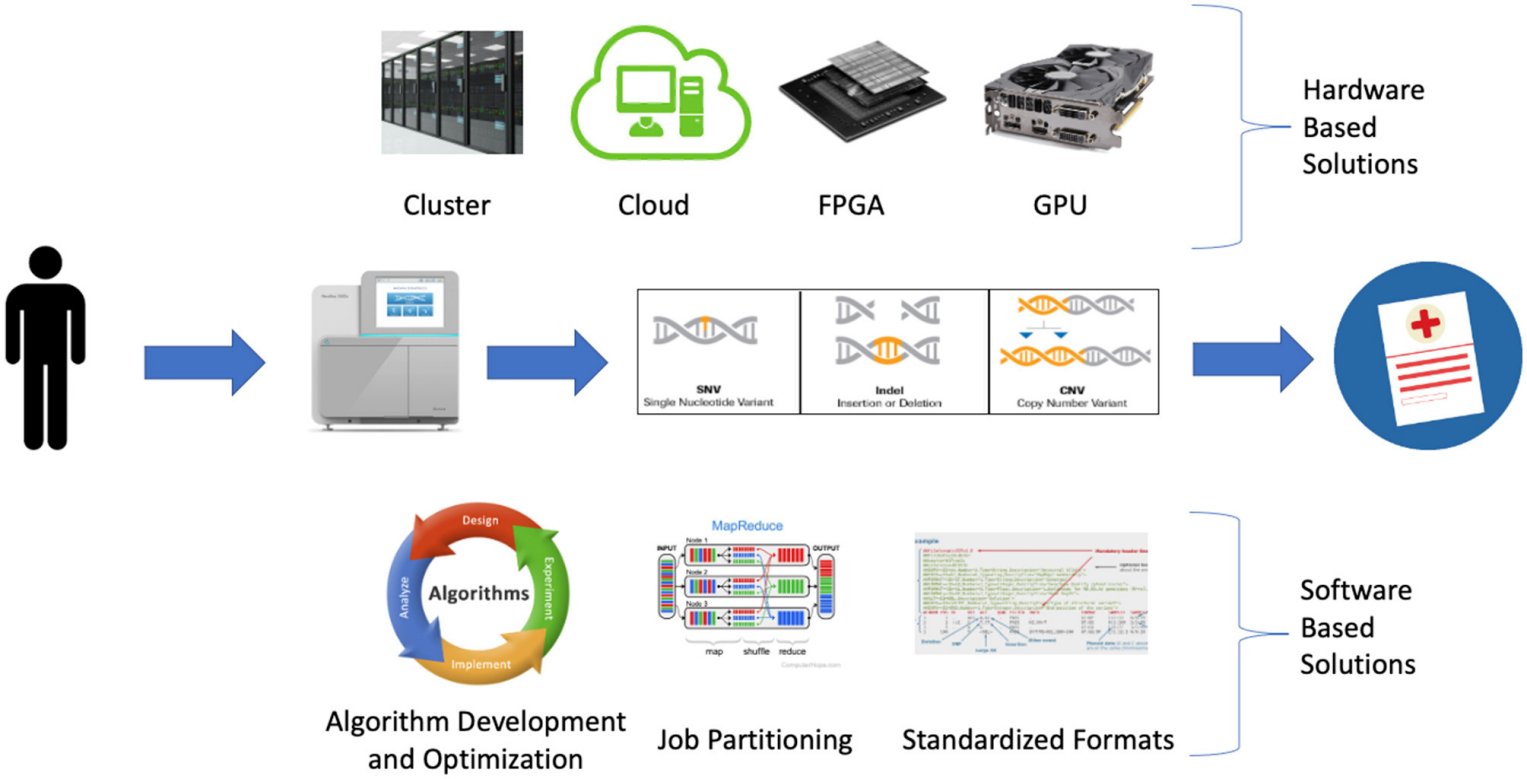
Software and hardware-based strategies being employed to address bioinformatic bottlenecks in large scale precision medicine programs.

A wide variety of strategies are being employed to detect clinically relevant genetic variation at scale. These approaches can be broadly classified as software-based or hardware-based (Figure 1).

### Software Based

Software development and optimization play an important role in improving precision medicine programs by improving algorithm performance and reducing run time and memory requirements. This is occurring *via* a variety of mechanisms including the development of new algorithms, optimization of existing algorithms, increasing parallelization *via* job partitioning, and standardized file formats (Table 2).

**Table 2:** Software based solutions.

**Strategy**	**Advantages**	**Disadvantages**
Algorithm development	– Develop novel approaches	– Requires community uptake
	– Existing suite of tools available for benchmarking	– Challenging to significantly change existing workflows
Algorithm optimization	– Quicker to improve existing algorithms	– Gains are often minimal if software well-designed initially
	– Simple to benchmark versus previous releases	– Any changes in expected output requires verification
Job partitioning	– Increases parallelization and reduces serial run time	– Splitting and combining results adds software complexity
Standardized file formats	– Standardized formats allows easy algorithm benchmarking	– No flexibility for new data types or information

New algorithms are being developed for a variety of analysis steps in variant detection workflows, particularly for variant prioritization. While the generation of either germline or somatic raw variant calls is increasingly routine [e.g., BWA for short read alignment (42) followed by GATK best practices (43)], development of algorithms to identify clinically relevant variants from raw variant calls remains an active area of software development. The increasing availability of variant annotation data has led to the development of annotation aggregator packages such as ENSEMBL Variant Effect Predictor (VEP) (31) or ANNOVAR (33). With external annotation sets and gene models rapidly updating, such tools are indispensable for applying the latest annotations to raw variant lists. Another area of active software development is predicting the functional impact of variant classes such as missense mutations. Heavily used tools such as PolyPhen2 (36) have been shown to exhibit high false positive rates (5) and newer tools are increasingly utilizing machine learning (38) and a consensus-based approach (44) to try to overcome these limitations however more work is needed to improve their accuracy. The most active area of development currently is disease-specific solutions with the increasing recognition that any disease requires tailored annotation / prioritization and may even require different types of sequence data. For example, with autoimmune disease T-cell receptor (TCR) and B-cell receptor (BCR) repertoires are often sequenced requiring custom software to identify the relevant clonotypes (45). Additionally, incorporating disease specific annotations [e.g., Immgen for autoimmune disease (46)] requires custom handling as disease-specific databases are generally not available within the annotation aggregation tools.

Ongoing development of many commonly used bioinformatics algorithms is reducing run time and memory requirements. For example, an update to the popular amplicon cluster software Swarm reduced memory usage by 50% and run time by 7X (47). These improvements are often driven by increasingly large data sets with many long-running software packages having been created when sequence data sets were smaller. Increasingly, individuals not involved in the original development of the software are finding ways to speed up and reduce memory usage of many commonly used algorithms. For example, an external group modified the popular Minimap2 (48) long read aligner by incorporating multi-index merging which reduced memory usage by an order of magnitude (49). While gains have been significant in many instances, further reductions in run time and memory usage will greatly facilitate the wide-spread uptake of precision medicine programs.

A common approach to reduce run time is increasing parallelization *via* programming models like MapReduce (50). MapReduce is a general purpose model designed to run efficiently over large datasets on commodity compute clusters. The incorporation of MapReduce by Apache Hadoop has led to its incorporation throughout the bioinformatics landscape, now found in software such as GATK (43) and BLAST (51). In addition to using models like MapReduce, custom solutions are often employed such as partitioning long-running whole genome jobs into smaller genomic chunks, often at the level of chromosome (52). Using this approach, we can expect at least an order of magnitude reduction in run time as the largest single chromosome represents <10% of the total human reference genome size. It should be noted that while this approach is suitable for algorithms where each chromosome is analyzed independently, this approach won't work when information from multiple chromosomes is required for an analysis (e.g., genome wide stats, detecting inter-chromosomal translocations). Another issue with this approach is the increased complexity required to manage the jobs and merge the per-chromosome output files.

File formats are increasingly being standardized to improve reproducibility and data sharing. For example, virtually all short read aligners now generate SAM alignment files while most variant detection software outputs variant call format (vcf) files. Standardizing file types can reduce ongoing storage requirements *via* improved compression which allows algorithms to work entirely with compressed data such as gzipped FASTQ files or compressed SAM files (BAM/CRAM). For example, read alignment generates an extremely large SAM file containing one row per read pair. Given whole genome datasets routinely contain >1 billion read pairs SAM files quickly become large and unmanageable for manipulation. To address this, a lossless binary version of the SAM file was created that reduces the file size by up to 75%. The resultant BAM file is significantly smaller and can be effortlessly queried and manipulated *via* bioinformatic packages such as SAMTools (53). Despite the improvements with BAM files, a more compressed format called CRAM was subsequently developed, resulting in a further reduction of 40–70% in size relative to BAM (54). While promising, a limitation of the CRAM format is that compression is not a lossless conversion whereas BAM compression is lossless. Overall file standardization has made significant improvements to variant detection workflow efficiency and portability however challenges do exist with frequent version changes often failing to maintain backwards compatibility.

### Hardware Based

Hardware developments are making significant contributions to precision medicine programs *via* increasingly large and accessible compute infrastructure and hardware accelerated solutions designed to address software bottlenecks. The increase in available computational resources is primarily driven by increasingly large and accessible cluster and cloud compute resources while the hardware accelerated solutions consist largely of new FPGA devices and GPU-enabled algorithms. Collectively the increasing uptake of these hardware-based solutions is easing existing computational bottlenecks within precision medicine programs (55). However while these hardware solutions are often designed to address the same bottlenecks, they differ with regard to ease of use, cost, performance and scalability (Table 3).

**Table 3:** Hardware based solutions.

**Resource**	**Advantages**	**Disadvantages**
Compute cluster	– Low cost entry	– Controller is single point of failure
	– Uses commodity hardware	– Technical expertise required
Cloud compute	– Highly scalable	– Data transfer and cost
	– No local installation	– Privacy concerns for sensitive data
FPGA	– Direct hardware / software link	– Challenging to program/re-program
	– Relatively low cost	– Integration requires technical expertise
GPU	– Cheaper than CPUs	– Chipset specific coding required
	– High parallelization possible	– Higher power usage than FPGAs

Most high throughput genome analysis workflows were originally designed to run on commodity clusters due to their affordability, scalability and relative ease of use. From small clusters running on local infrastructure to enterprise-level systems with thousands of readily-available cores, their design follows the same model with scheduler software responsible for managing jobs and resources across a distributed system of linked computers. This setup enables efficient parallelization of jobs using commodity hardware with minimal overhead. As such systems grow with more users and resources however, increasing levels of expertise are required for seamless operation. With such expertise, clusters are able to process huge numbers of jobs in parallel making this infrastructure critical to many project requiring efficient and timely data processing.

In addition to increasingly large compute clusters, accessible and expandable cloud-based compute resources are driving an increasing number of precision medicine programs (56). In contrast to cluster based solutions, cloud solutions perform all analyses on remote systems across a network connection. In a cloud based model, storage and compute resources are commodities that can either be borrowed or rented from a provider such as Amazon Web Services or Microsoft Azure. The greatest advantage of cloud compute is its flexibility; users can access exactly the resources required for virtually any job. This flexibility enables users of any size to utilize cloud resources providing the appropriate compute environment is available. Setting up custom cloud-ready workflows requires a significant effort initially although increasingly the most common genomics workflows are being made available [e.g., nf-core (57)]. Potential issues with public cloud resources include issues handling sensitive patient information and challenges moving large genomic data sets. To address these, some groups are opting for a hybrid solution by creating private cloud infrastructure potentially getting the benefits of both cluster and cloud approaches. Regardless of the approach, it is clear cloud compute infrastructure will play an increasingly large role in precision medicine programs (16).

Beyond increasingly large and flexible compute infrastructure, hardware accelerated solutions such as GPU-enabled algorithms and FPGA devices are now being used to reduce run time in precision medicine programs (15). While GPU-enabled versions of many popular bioinformatics algorithms have existed for a long time [e.g., GPU-BLAST is 10 years old (58)], it is only recently that we are beginning to see wide-spread uptake of these algorithms. Algorithms able to utilize GPUs can significantly increase parallelization by taking advantage of the large number of specialized cores on a single graphics card. In contrast with sequential CPU processing, GPUs offer superior scalability and reduced costs per unit however the biggest challenge is creating the specialized code required to utilize GPUs. Further, portability is a challenge as any GPU code developed is vendor-specific meaning it cannot run on another vendors GPUs. In reality, most GPU-enabled bioinformatics algorithms are currently written using NVIDIA's Compute Unified Device Architecture (CUDA) with examples from variant detection workflows focused on the short read alignment step [e.g., SOAP3 (59)]. However, with the increasing availability of GPU-enabled algorithms across the whole research spectrum more options relevant to precision medicine are likely forthcoming.

In addition to the potential of GPU-enabled algorithms, an increasing number of FPGA devices are available for precision medicine variant detection (60). FPGAs are integrated circuits designed to be configured for specific software applications. FPGAs offer many advantages in that they are flexible, inherently parallel, re-programable and relatively low cost. The greatest limitation of FPGA is they are very difficult to program compared to GPUs (15) however devices exist for both short read alignment [Bowtie (61)] and even entire precision medicine workflows (DRAGEN). Developed by Edico Genome and now owned by Illumina, DRAGEN can reduce already parallelized variant detection workflows by up to an order of magnitude (62). DRAGEN has also been deployed at scale in partnership with Genomics England for their rare disease analysis platform. It is clear FPGA devices have a significant role to play in precision medicine.

While all the software and hardware solutions are described in isolation, in reality various hardware and software combinations are being tested in new precision medicine workflows.

### Variant Detection

Detecting small genetic variants within sequenced human genomes is a relatively mature high-through sequencing application. Despite this progress, challenges remain to comprehensively characterize all variation. Variant detection challenges include an incomplete human reference genome, a limited number of robust validated variant truth sets and no clear best performing algorithm; challenges which are amplified for less well characterized variant classes such as repeat and copy number variation which are increasingly being implicated in human disease (63).

Since the initial human genome assembly in 2001, improvements in both software and long read sequencing technology have improved the genome assembly to the point where we now have the first telomere-to-telomere genome assembly for most chromosomes (64). While promising for the future, most precision medicine programs currently utilize the GRCh38 assembly and will likely continue to do so for the near future largely due to the abundance of well characterized annotations data reported relative to these genomic coordinates. For example, one of the most important annotation sets GNOMAD (28) only converted to GRCh38 in October 2019, almost five full years after the initial GRCh38 assembly was released in December 2013. A similar period of time will likely be required to convert existing workflows and annotations to the improved telomere-to-telomere assembly following wide-spread acceptance within the community. For context, the GRCh38 assembly still contains 850 sequence gaps with numerous mis-assembled regions reported over the years.

Improving variant detection workflows requires robust validated variant truth sets for benchmarking both new algorithms and updated versions of existing algorithms. Until quite recently a single reference dataset (NA12878) was available for benchmarking which was limited by ~30% of the reported variants being classified as low confidence due to either low coverage, local alignment problems, or systematic sequencing errors (65). The wide-spread availability of high quality long read sequence data and the increased number of samples available within consortiums like Genome in a Bottle mean an increasing number of relatively complete high quality variant truth sets are available for benchmarking.

While the algorithms for detecting SNVs and small indels are increasingly accurate and reliable, the algorithms for detecting other types of variation such as repeat, copy number and structural variation remain an active area of development. To illustrate, a recent review reported SNV and small indel F-scores of >0.975 and >0.85, respectively, (12) while a review of copy number and structural variant detection algorithms reported precision values of between 0.40 and 0.91 and recall values from 0.07 to 0.28 depending on the type of variant being detected (66). Limited data is available reporting the true accuracy of repeat variation detection algorithms due to lack of a gold standard reference validation set with most tools instead relying on analyses using *in silico* data. It should be noted that despite the highly precision and recall reporting for SNV calling, studies have shown that recurrent false positive variants are routinely called and exist within variant repositories (67).

Central to any analysis step is the selection of the algorithm(s) to run. While for many analysis steps a single algorithm is determined to perform sufficiently, for many variant detection applications leading algorithms generate highly discordant results with no single algorithm performing optimally under all conditions (52). To address this, an increasingly popular approach is to run multiple algorithms and apply a consensus approach in order to minimize the effect of any potential biases within a single algorithm [e.g., DNA (52)/RNA (68)]. This approach has been shown to generate the highest quality variant data sets for either specificity or sensitivity depending on whether the intersection or the union of the variant calls is taken, respectively.

### Variant Interpretation

Whole genome sequencing (WGS) generates millions of raw variant calls, the large majority of which are not relevant to disease. While targeted sequencing experiments such as exome or gene panel sequencing reduce the number of raw variant calls, the challenge of variant filtering and interpretation to identify clinically relevant variants remains. Beginning with raw variant calls, the most common filtering strategy is to apply a series of successive annotation and prioritization steps in order to reduce the genomic search space for clinically relevant variants. Such strategies include stratifying variants by impact on genes, running functional inference prediction software for missense mutations, overlapping to both ethnically matched population-level and disease-specific variant repositories, and sequencing pedigrees for germline disease and paired tumor/normal samples for cancer (Table 4). Overall, each step reduces the genomic search space with an overarching goal of reducing the final list of candidate variants down to a size suitable for in-depth manual interrogation.

**Table 4:** Strategies for variant prioritization.

**Strategy**	**Strengths**	**Limitations**
Consensus-approach running	– Minimize algorithm biases	– Adds computational complexity
multiple algorithms	– Reduce specificity or sensitivity by taking intersection or union	– Longer run time
Stratify by impact on genes	– Prioritize disease enriched variant	– Changes reported relevant to specific version of gene model
	sets (e.g., missense or splice-site variants)	– Multiple isoforms often available
Functional inference prediction	– Prioritize mutations likely to disrupt protein	– Tools have known high false positive rates
software		
Overlap population-level	– Allows filtering of common population-level variation	– Contains errors and incomplete records due to lack of curation
variant databases
Overlap disease-specific	– Identify variants or genes previously implicated in disease	– Large numbers of non-causal variants often included
databases		
Pedigree sequencing	– Generate pedigree-wide annotation (disease inheritance	– Obtaining samples for larger family
	compound heterozygosity, etc)	
Paired cancer sequencing	– Matched tumor/normal samples can detect somatic variation	– Sample purity
		– Tumor heterogeneity

Often the first annotation step is to stratify variants based on their impact on genes. For example, SNVs causing non-synonomous/nonsense mutations or small indels situated within exons causing a frameshift are prioritized. Determining this impact can be challenging however due to factors such as differences in gene models or multiple isoforms reported within a single gene model. For example, a recent study aligned RNA-Seq data to three popular gene models (ENSEMBL, RefSeq, and UCSC) and found 95% of non-junction read alignments were identical across the three gene sets however only 53% of junction spanning read alignments were identical (69). Such studies illustrate the importance of careful gene model selection. Even within a single gene model multiple isoforms are often reported, meaning the choice of isoform can alter the expected impact on the gene. Many workflows opt to compare the impact across all isoforms and report the most severe outcome while others report the impact relative to the annotated “canonical” transcript as reported by gene models such as ENSEMBL, RefSeq, and UCSC.

Another challenge in variant interpretation is the identification of missense mutations most likely to disrupt protein function. With hundreds or thousands of missense mutation calls per patient, a large number of computational tools have been developed to prioritize these variants. Such tools are generally trained on validated disease mutations as a positive set and common polymorphisms as a negative set and consider three main types of evidence; sequence conservation, protein structure, and protein annotations. These tools however are untested against the full spectrum of random *de novo* mutations and validation studies have reported consistently high false positive rates for both candidate disease-causing (5) and pharmacogenetic variants (21). Increasing gains in performance are reported by tools that apply a consensus approach by incorporating scores from other algorithms into their own scoring (e.g., CADD (38). Additional gains have recently been reported in algorithms applying machine learning approaches trained on increasing large data sets (70) however wide spread validation studies are required to validate these claims.

Databases of population-level variation are extremely valuable for reducing the search space *via* the removal of common variants as candidates. Databases like dbSNP (27) and GNOMAD (71) contain increasingly detailed population-level variant frequency information which allows both the de-prioritization of common variants as well as the prioritization of rare or *de novo* variants. It is critical when applying such filters to use ethnically matched allele frequencies using the increasingly granular variant information available within the variant repositories. Without ethnic matching, many variants are incorrectly characterized as novel or rare due to under-sampling in the repository of the patient's ethnic group. Despite efforts in recent year to increase numbers of under-represented ethnicities in such databases, much work is needed to include all groups such as Indigenous populations (72).

Equally important to population-level databases are human disease databases which allow previously implicated variants and/or genes to be prioritized. Databases of clinically relevant variants are numerous and growing rapidly in size (e.g., ClinVar (34) for germline and CIVIC (2) for cancer). Importantly, these databases follow standardized Human Genome Variation Society (HGVS) approved nomenclature for DNA and RNA variants allowing direct comparison across disparate data sets. In addition to comprehensive generic disease databases, increasingly disease-specific databases are being developed such as Infevers (73) for auto-inflammatory disorders or IARC TP53 (74) for TP53 specific mutations. While disease databases are an extremely valuable resource, most have been shown to contain high numbers of false positive due to manual curations being made with incomplete functional data. For example, one study found 27% of reported recessive disease-causing variants were false positives and were actually either common polymorphisms or mis-annotated (25). Such studies highlight the need to improve such databases *via* increasingly rigorous functional validation studies.

A powerful approach for reducing the search space for disease-causing variants in rare disease is the sequencing of families or pedigrees. Using this approach there are two main applications; sequencing trios with an affected child and two unaffected parents or sequencing multiple members of larger pedigrees containing multiple affected members. In both instances custom software is required to identify the variants most likely to be causal; namely *de novo* mutations in the trios and variants shared between affected and missing in unaffected members in the larger pedigrees. With pedigrees, specialized software is required to concurrently consider all variation and provide pedigree-specific annotation such as disease inheritance patterns, phasing information, and potential compound heterozygosity (75). While such tools are increasingly mature, more is needed to incorporate their results into precision medicine workflows.

For detecting somatic mutations in cancer, the most effective strategy is sequencing paired tumor and normal samples and analyzing them simultaneously with cancer-specific software to identify candidate driver mutations (76). The presence of a matched control sample facilitates the identification of somatic variants however issues such as sample cross-contamination and tumor heterogeneity ensure cancer-specific software is required for reliable somatic variant detection. In this space, single cell sequencing has the potential to mitigate some of the issues around sample heterogeneity (77).

While currently most precision medicine programs run some combination of the above annotation steps in series, increasingly machine-learning based approaches are being developed to identify clinically relevant variants directly from raw variant lists (78). While much work is required to achieve this lofty goal, machine-learning based approaches are already being used successfully for more specific applications within the larger workflows such as detecting variant pairs causing disease (79), prioritizing non-coding variants (80) and identifying new pharmacogenetic variants (22). While these applications show promise, to date there are limited examples of large machine learning approaches being utilized at scale in precision medicine programs (81). In fact, a recent review could identify only a few examples of machine learning methods impacting clinical practice; an observation they largely attributing to the poor performance of the predictive models, difficulties interpreting complex model predictions and lack of validation in clinical trials sufficiently demonstrating improvements to current standard of care (82).

## Discussion

Precision medicine programs continue to mature and expand around the world (1). One of the most common application in such programs is detecting genetic variation relevant to a patient's condition. Significant improvements in both software and hardware over the last few years have made the detection of small genetic variation from patient sequence data an increasingly routine process. To improve the success of existing programs, work is required both with regard to detecting large and repetitive genetic variation routinely and with improving the automation of variant prioritization. In the near-future, it will also be critical to synthesize patient clinical data with a variety of sequence data types.

Repeat variation is broadly classified as mobile elements and tandem repeats which are further divided by size in short tandem repeats and satellites. Due to challenges detecting repeat variation using short read sequencing their frequency is largely unknown but current estimates are ~10,000 tandem repeats and ~2,000 mobile elements per human genome (83). Repeat variants are important as they are increasingly being implicated in driving human disease, particularly neuropathological disorders like autism (84). Similarly larger structural and copy number variation (generally defined as deletions, insertions, duplications, inversions and translocations >50bp) are increasingly being cataloged and implicated in driving disease, particularly in cancer (85). Despite the importance of these variant classes to human disease, they are largely not being interrogated in current precision medicine programs due to challenges detecting them. To address this, substantial work is needed in several areas including improved detection algorithms, better validation truth sets and repositories of both population-level and clinically-relevant variation. Long read sequencing will play a critical role in generating these improved repositories and truth sets.

While variant interpretation and prioritization workflows continue to improve, greater automation of the process is required to alleviate this current bottleneck. While annotation aggregators like VEP are continually incorporating additional external data sets, custom workflows are typically still required to collate and rank variants most likely to be clinically relevant. The desired output of such a workflow is a small list of candidate variants suitable for manual interrogation which will undergo an in-depth investigation for potential inclusion in the final clinical report. This manual process is extremely time-consuming however and requires further automation. While challenging to automate, software is urgently needed which inputs a raw vcf file and the relevant clinical information and outputs a small lists of likely causal variants suitably annotated for a clinical report. While an increasing number of groups are tackling these problems, more work is needed.

While currently most programs focus on detecting genetic variants using short-read DNA-based sequencing (e.g., targeted gene panels, exomes or WGS) increasingly other patient sequence data is being generated including transcriptome, long read, microbiome and single cell sequencing. For example, sequencing the transcriptome from a patient can be used to identify transcriptional changes likely caused by genetic mutations. A recent study used this strategy to improve diagnosis rates by 35% over genome sequencing alone by identifying deep intronic variants which altered splicing (13). Long read sequencing is increasingly being employed to detect complex variation unable to be easily detected with short read technologies (86). If the cost and quality of long read sequencing continues to improve it is feasible that long reads can be used routinely in precision medicine programs in the future. Microbiome is likely to be important in future programs as well. Dysbiosis of the microbiome is increasingly linked to human disease and the ability to examine differential abundance of metagenomic data (87) before and after treatment represents a new avenue for exploration (88). Finally, single cell sequencing technologies will have an increasingly large role to play given their ability to detect disease causing variants at single cell resolution over time (77). While such possibilities are exciting, it is clear current workflows are unable to work with complex multi-omics patient data sets and that substantial developments in software and hardware are required to support this in the future.

## Future Challenges

The ongoing success of precision medicine programs for genetic disease has led to increasingly large and diverse sequence information being generated per patient. Programs are expanding in terms of number of patients sequenced, the sequencing technology employed and the type of diseases being examined. Scaling up and standardizing existing programs to population level numbers requires significant improvements in the throughput and interoperability of the systems. The other significant challenge will be the incorporation of information from additional sequencing applications including transcriptome, long read, microbiome, and single cell sequencing. The next generation of supporting software and hardware needs to be flexible and robust to manage the coming deluge of data.

## Conclusion

Identifying clinically relevant genetic variation is one of the hallmarks of successful precision medicine programs. This review discusses the wide variety of strategies being employed to both speed up and improve the detection of clinically relevant variants. While challenging today, increasingly complex patient data sets will be generated in the near future which will require sophisticated hardware and software solutions. To support this, substantial new methodologies able to synthesize large volumes of disparate data types will be needed. These new tools will allow precision medicine programs to realize their full potential both now and into the future.

## Author Contributions

The author confirms being the sole contributor of this work and conceived and wrote the manuscript. The author approved it for publication.

## Funding

MF is funded by NHMRC APP5121190.

## Conflict of Interest

The author declares that the research was conducted in the absence of any commercial or financial relationships that could be construed as a potential conflict of interest.

## Publisher's Note

All claims expressed in this article are solely those of the authors and do not necessarily represent those of their affiliated organizations, or those of the publisher, the editors and the reviewers. Any product that may be evaluated in this article, or claim that may be made by its manufacturer, is not guaranteed or endorsed by the publisher.
